# Book Notices

**Published:** 1888-11

**Authors:** 


### Book Notices.

A Text-Book of Human Physiology; by Austin Flint, M. D., LL. D. Seven hundred and ninety pages, three hundred and sixteen figures and two plates. Fourth edition, entirely re-written. D. Appleton & Co.

The elaborate description of apparatus and methods as well as the numerous historical references contained in former editions of this work, have been left out and the entire volume re-written, which makes it practically a new book.

The author has adopted the new chemical nomenclature, and has included both the English weights and measures and the metric system. A number of new plates and figures have been introduced, and a great many have been discarded. In fact, all the defects of the former editions have been corrected and all the recent advances in physiology have been duly noticed. We risk nothing in saying that the work, as it stands, is perhaps the best text-book published.

---

Diseases of Women. The Pathology, Diagnosis, and Treatment, by Graily Hewitt, M. D., London, F. R. C. P., Professor of Midwifery and Diseases of Women, University College, and Obstetric Physician to the Hospital, etc., etc. A new American, from the fourth revised and enlarged London edition, with 236 illustrations, edited with notes and additions by H. Marion-Sims, M. D., New York. Three volumes, of 350 pages each; published by E. B. Treat, 771 Broadway, N. Y., 1887, $2.75 each.

This is a standard work on the diseases peculiar to women, gotten up in the best of style, with clear, open type, and well illustrated with numerous figures of the most practical character. The most striking position taken by the author is that "the changes in the shape and position of the uterus are directly or indirectly resposible for all the sufferings and discomforts attendant on the affections peculiar to the female sex." He accounts for reflex vomiting during pregnancy by "changes in shape and position of the uterus," and claims certain relief by treatment which removes these pathological conditions.

It is a work worthy of a large sale. The publisher has done his part well.

---

THE PHYSICIANS' POCKET DAY BOOK. Designed by C. Henry Leonard, A. M., M. D. Size, 7½ inches long, 3½ inches wide and ⅜ of an inch thick. Bound in red morocco, for the pocket; pencil loop and flap, red edges. Price $1.00 postpaid. The Illustrated Medical Journal Co., Publishers, Detroit, 1888.

This is the 10th year of issue of the exceedingly popular Day Book, which contains several new features. Besides accommodating daily charges for thirteen months for fifty families, and the other usual memorandum pages, it has a very complete list of doses of Old and New Drugs; Poisons and their Antidotes, Tried Tests of Urinary Deposits, Chemical and Microscopical; Obstetric Calendar; Disinfectants for the Sick Room and Vaults; Tables of Weights and Measures; Table of Eruptive Fevers, and Drops in a drachm of fluid medicine.

---

ANNUAL OF THE UNIVERSAL MEDICAL SCIENCES.—A yearly report of the progress of the general sanitary sciences throughout the world. Edited by Charles E. Sajous, M. D,, lecturer on laryngology and rhinology in the Jefferson Medical College, Philadelphia, and seventy associate editors, assisted by over 200 corresponding editors, collaborators and correspondents. Illustrated with chromos, lithographs, engravings and maps. F. A. Davis, publisher, Philadelphia, 1888.

Ths work for 1888 is to be in five volumes of about 500 pages each, and judging from the first two volumes which we have had time to examine, they are all the editor claims for them, and should be in the library of every progressive physician. All that is new bearing upon all medical subjects the world over during the past year is arranged and condensed in the most approved style, We heartily commend this publication and bespeak for the enterprising publishers a most extensive sale.

---

TRANSACTIONS OF THE STATE MEDICAL SOCIETY OF ARKANSAS, held at Fort Smith, April, 1888.

The volume contains 124 pages, well filled with good matter, in small clear type. Membership about 200, represented mainly through county societies. This meeting of the State Society distinguished itself by passing certain resolutions against the eacouragement charletanism receives at the hands of the religious press. May the war continue.

---

PROCEEDINGS OF THE FLORIDA MEDICAL ASSOCIATION. SESSION OF 1888.

This is a neat pamphlet of eighty-four pages, and in general interest is quite an improvement over the Transactions of 1887.

The paper by Dr. C. J. Kenworthy, of Jacksonville, entitled "Yellow Fever Lessons," raised a ripple in the Association. Dr. Kenworthy thinks there is too much red tape business about Surgeon-General Hamilton to suit the people in Southern Florida. This paper is replied to by Dr. J. V. Porter, in a manner calculated to do much damage to Dr. Kenworthy's assertions.

A number of other very worthy papers appear in the Proceedings, principally upon the subject of yellow fever.

---

APPLIED ANATOMY OF THE NERVOUS SYSTEM, being a study of this portion of the human body from a standpoint of its general interest and practical utility in diagnosis, designed for use as a text-book, and a book of reference. By AMBROSE L. RANNEY, A. M.; M. D., Professor of the Anatomy and Physiology of the Nervous System in the New York Post-Graduate Medical School and Hospital, etc. 772 |pages, 2nd edition re-written, enlarged and profusely illustrated. D. Appleton & Co., 1888.

This work, in the first edition, has been tested and adopted as a text-book throughout the country. When this much can be said of a book, little else need be added. It is a standard work, and that commends it to the public.

The author truthfully says: "When we consider that every act, which distinguishes the animated being from the corpse, is dependent upon the influence of the nerves, and that, without these electric wires, the heart would cease to throb, the lungs no longer perform their functions, the eye no longer be capable of vision, the ear no longer perceive sound, and that smell, taste, expression and movement would cease to exist, we can then understand how much of physiological interest must center around this special study, and how necessary is the thorough understanding of the distributlon and function of the individual nerves, if we ever hope to attain a comprehensive grasp of the general plan of our construction."

